# Assessing the Extent of Adherence to the Recommended Antenatal Care Content in Malaysia: Room for Improvement

**DOI:** 10.1371/journal.pone.0135301

**Published:** 2015-08-13

**Authors:** Ping Ling Yeoh, Klaus Hornetz, Nor Izzah Ahmad Shauki, Maznah Dahlui

**Affiliations:** 1 Department of Social and Preventive Medicine, Faculty of Medicine, University of Malaya, Kuala Lumpur, Malaysia; 2 German Development Cooperation, Berlin, Germany; 3 Family Health Development, Selangor State Health Department, Shah Alam, Malaysia; 4 Mediconsult Sdn. Bhd., Ampang, Malaysia; TNO, NETHERLANDS

## Abstract

**Background:**

Recent papers on monitoring of health services affirmed that while antenatal care (ANC) is an effective measure, quality is still a problem. Quality in maternal services “…involves providing a minimum level of care to all pregnant women…” Yet adherence to a minimum level of recommended ANC content appears to be unmet. Comprehensive review of ANC content rendered in environments with rapid changes in demographic, socio-economic, lifestyle and morbidity was sparse. Malaysia is such a country that has undergone these transitions with tremendous progress in health. However, recent progress in pregnancy outcomes is stagnating. This study aims to analyse adherence to recommended ANC; specifically, to examine the extent of adherence to recommended ANC content and to determine the factors influencing ANC content score.

**Methods:**

A retrospective cohort study of 522 randomly selected women who used ANC was conducted. Data were extracted from individual records. The study examined adherence to essential ANC guidelines using weighted scoring for physical examination, health screening, case management, and health education. GLM Univariate analysis procedure was used to determine the factors associated with ANC content score. Binary logistic regression was used to assess ANC content level and pregnancy outcomes, controlled for ANC utilisation.

**Results:**

Around half of the women had <80% of recommended ANC content documented. Health education had the lowest mean score, at around 35%. The low-risk pregnancies had a higher ANC content score than the high-risk pregnancies (78% vs. 75%; P = 0.002). The smallest clinics had a higher ANC content score than the bigger clinics (78% vs. 74–76%; P<0.001). ANC content score among the women with “adequate” ANC utilisation, as defined by the modified Adequacy of Prenatal Care Utilisation Index, was lower than the women with “adequate-plus” ANC utilisation (75% vs. 78%, P<0.001). Assessment of symphysis-fundal height, foetal presentation and foetal heart auscultation were initiated earlier than recommended. Inadequate ANC content was associated with higher prevalence of preterm birth.

**Conclusions:**

Our findings indicate the presence of issues related to delivery of recommended ANC content. We advocate for all pregnant women to be ensured of adherence to the recommended ANC content. We also recommend monitoring the delivery of health advice. Conforming to recommended timing of initiation for ANC practices is essential due to resource implication and possible implication on maternal wellbeing. The association of inadequate ANC content and preterm birth may be due to lesser opportunities to receive some of the care because of lower number of ANC visits among preterm birth; this may also indicate the importance of having adequate ANC content.

## Introduction

Recent strategic papers on monitoring of health services affirmed that while ANC is an effective preventive measure, quality of care is still an issue that requires additional monitoring and evaluation. [1, 2]. Quality of care in maternal services “…*involves providing a minimum level of care to all pregnant women and their newborn babies and a higher level of care to those who need it…”* [3]. Yet adherence to minimum level of recommended content for antenatal care (ANC) appeared to be unmet. Studies assessing the extent of adherence to minimum level of recommended ANC content revealed that majority were unable to meet the national standards in the developing countries [4–6], and also in developed settings [7–9]. Physical assessment and basic laboratory screening had higher compliance than health education or other prescriptions [4–7, 9].

There has been few recent reviews on comprehensive aspects of ANC content rendered in environments with rapid changes in demographic, socio-economic, lifestyle and morbidity [5, 10, 11]. Malaysia is such a country that has undergone immense transitions over the past decades. At the same time, Malaysia has made tremendous progress in health status and healthcare [12], as well as reached excellent maternal-child-health services coverage [13, 14]. Recent progress in pregnancy outcomes however is stagnating. Birth weight under 2,500g was reported at 11% of total live births, higher than some neighbouring countries [15]. Stillbirths was 6 per 1,000 total births, doubling that of developed nations [14]. In addition, maternal mortality ratio remained stagnant at around 28–30 per 100,000 live-birth since over a decade [16, 17]. Reasons for these stagnation need to be analysed and policy options for change be identified.

This study attempted to contribute to this end. It aimed to analyse adherence to recommended ANC, specifically: (i) to examine the extent of adherence to recommended ANC content; and (ii) to determine the factors associated with ANC content from the obstetrics, provider and utilisation perspectives.

### Background of study setting

Healthcare in Malaysia operates a dual healthcare system consisting of a tax-funded government-run universal healthcare system and a private healthcare system [12]. The Ministry of Health (MOH) offers a comprehensive range of services through primary care health clinics and hospitals [12]. A primary care health clinic is planned to serve 15,000 to 20,000 population. It is staffed by doctor, dentist, pharmacist, assistant medical officer, public health nurses, assistant pharmacy officer; delivering services such as general outpatient care, dental care, maternal and child care, health promotion and family planning [12]. Malaysian health system is considerably centralised and uniform. All the 16 states/ federal territories in Malaysia shares similar organisation of health services delivery and care protocol including ANC, especially among the states in Peninsula Malaysia.

The World Health Organization (WHO) recommends a minimum of four ANC visits during pregnancy. The recommended interventions for improving maternal health in the pregnancy period are classified as: (i) routine care offered to all women and babies, (ii) additional care for women and babies with moderately severe diseases and complications, and (iii) specialised obstetrical and neonatal care for women and babies with severe diseases and complications [18]. The routine care offered to all women aims to monitor and improve the wellbeing of the mother and foetal, detect problems complicating pregnancy (e.g. anaemia; hypertensive disorders; bleeding; mal-presentation; multiple pregnancy), respond to other complaints, prepare for birth, and promote healthy behaviours.

The MOH Malaysia recommends ten ANC visits for normal uncomplicated primigravida and seven visits for normal uncomplicated multigravida based on a 40-week pregnancy. Recommended content for ANC at booking visit and subsequent routine care are defined in the guidelines [19]. Risk assessment is to be conducted during ANC visits at specific gestational periods [19]. The risk assessment classifies pregnant women into risk levels using colour coding system that functions as a managerial tool to determine the level of care required according to the condition of the pregnant women. This enables a midwife to refer pregnant women to a specialist unit without red-tape and assures that pregnant women with problem are being examined by a doctor or a specialist without undue waiting times [20]. The risk level is classified as (from no/ low risk to high risk):

- **White**—Pregnancy without risk factor, to be followed-up by nursing staff at health clinic,
- **Green**—Pregnancy with low-risk factor, to be referred to medical officer at health clinic for decision on subsequent provider (to be followed up by medical officer, or nursing staff at health clinic),
- **Yellow**—Pregnancy with high-risk factor, to be referred to the Obstetrics and Gynaecology specialist at hospital or Family Medicine Specialist at health clinic within 48 hours,
- **Red**—Pregnancy with extreme high-risk factor requiring urgent medical attention, to be referred to hospital immediately.

## Methods

### Study design and population

This is a retrospective cohort study of women who were pregnant and used ANC at selected primary health care clinics. Data on ANC visits were extracted from individual ANC records. Study sites included six out of 58 public sector primary health clinics in Selangor state, Malaysia. A total of 522 eligible records were analysed. Multistage random sampling was applied, using type of health clinics by their planned daily workload as the stratification factor. This involved two separate stages:

- Stratified all health clinics of Selangor into three strata by planned daily workload: below 150, 150–300, and 301–500 and above. We then sampled two health clinics from each stratum (total six health clinics).
- Sampled pregnant women (ANC records) from each of the six health clinics according to proportional allocation based on the combined planned daily workload of each stratum (34%, 47%, and 19% in the order mentioned above), and the estimated proportion of pregnant women by risk level (30% white-tagged and 70% coloured-tagged). The eligibility criteria were:
  - Inclusion criteria—Malaysian citizens; completed their pregnancy and delivered at gestational age ≥ 22 weeks; delivered in year 2013, January to June (±1 month).
  - Exclusion criteria—transfer-in cases from other health provider or clinic; transfer-out cases to other health provider or clinic; multiple pregnancies.

Ethical approval for the study was provided by the Research and Ethics Committee of University Malaya, Malaysia, and the Medical Research and Ethics Committee of Ministry of Health, Malaysia. Patient records/information was anonymized and de-identified prior to analysis.

### Antenatal care content components

Assessment on ANC content adequacy used weighted scores for physical examination (PE), health screening (HS), case management (CM), and health education (HE), based on the MOH guidelines for ANC [19]. The minimal level of recommended ANC content and the corresponding criteria for scoring is presented in S1 Table. Where applicable, the criteria for scoring considered factors such as gestational age of birth, gestation of initiation, and user behaviour. These were important because the extent of adherence to ANC content which had an implication on quality of services delivered would be inaccurate if for example, late initiation of ANC or low number of visits due to user default were not considered. The components of assessment (S1 Table), assignment of weighting factor and cut-points were established in consultation with a team of experts from the Family Health Development Unit of the State Health Department. The assignments were based on the following reasoning:

- The team was of the opinion that PE and CM are the two most important aspects, while HS is compulsory to support the clinical care.
- While HE is crucial, among all the components, the quality of HE is the least that could be standardised due to variants in terms of content, duration, delivery methods in which the information is not available in this retrospective study. Standardisation for HE in this study could only be based on whether a HE topic was covered. Therefore it is less relevant to assign heavier weightage for HE component.
- The team examined the possible combinations of weighting factors, the weighting of 0.30, 0.25. 0.30 and 0.15 in the order for physical examination (PE), health screening (HS), case management (CM), and health education (HE) respectively represented an optimal combination. This combination is able to establish a higher priority on PE and CM, followed by HS, and a reasonable lower weighting for HE as explained above.
- ANC content score is categorised as inadequate (≤ 79%) and adequate (80–100%). ANC content should score at least 80% of total scores to be considered adequate because the ANC content assessed represents the basic care that a woman should receive. Previous studies on ANC content had used the same cut-points [5, 7].

### Data extraction and variables description

In addition to ANC content rendered, we extracted obstetrics and providers covariates which we were interested in determining the association with content score. Dependent variable was the ANC content scores. Independent variables included: parity (nulliparous, multipara), risk level of pregnancy (low-risk and high-risk), clinic type by planned daily capacity of patients (below 150, 150–300, 301–500), percentage of total visits attended by specific providers (community nurse, registered nurse with postgraduate qualification, medical officer), and ANC utilisation adequacy. Risk level of pregnancy were derived from the risk factors assessment system [19]. Low-risk denotes pregnancies without risk or with only low risk factors (white and green tags), and high-risk refers to pregnancies with high risk factors (yellow and red tags).

ANC utilisation variable considered gestational age of first visit and observed-to-expected visits ratio which was adjusted for the gestational age at delivery, based on the concept of the Adequacy of Prenatal Care Utilisation Index (APNCU Index) [21]. This index was modified to reflect the lower ANC schedule in Malaysia compared to the original APNCU Index. The original APNCU Index was based on 13 recommended visits; in comparison, the Malaysian ANC guideline recommends ten visits for primigravida and seven visits for multigravida. The cut-points of the original APNCU index’s observed-to-expected visit ratio categories are: ≥ 110% (adequate-plus), 80–109% (adequate), 50–79% (intermediate), and <50% (inadequate). When this is applied to the local guidelines which has lower recommended schedule, one additional observed visit compared to expected visit will fall into the range of ≥ 110% (adequate-plus), presenting a bias commented by Koroukian and Rimm concerning the original APNCU Index [22]. On the other hand, one additional visit at the recommended visits of 13 which the original index based upon will still be within the 80–109% (adequate) range. The observed-to-expected visit ratio cut-points therefore were modified to accommodate the lower recommended visits of Malaysia guidelines. The modified ratio cut-points became: ≥ 130% (adequate-plus), 90–129% (adequate), 60–89% (intermediate), and <59% (inadequate). For analysis of the result, adequacy of utilisation was categorised into three categories: adequate-plus (denotes utilisation ≥ 30% higher than recommended visits), adequate, and inadequate (intermediate category was grouped with inadequate category).

The outcome measures used to assess the association with ANC content adequacy were preterm birth (PTB, less than 37 weeks of gestation at birth); low birth weight (LBW, less than 2,500g at birth), stillbirth (intrauterine deaths of at least 22 weeks gestation or over 500g weight), and the combined foetal outcomes.

Provider profile of the health clinics was captured based on the staffing records provided by the nursing officer of the health clinics. This inventory focused on providers who were regularly involved in the delivery of ANC, and who have directly interacted with the women during their ANC visits. The main cadre of providers that were used for data analysis of this study included community nurses, staff nurses with and without post-graduate training, medical officers, and family medicine specialist who could be either in-house or visiting (external) resource.

### Statistical analysis

ANC content scores were tabulated using the compliance criteria for scoring (S1 Table). Pre-assigned weighting factor was then applied to obtain the weighted scores for each component. SPSS Statistics Version 21 was used for all statistical analysis. We used GLM Univariate to examine the association between obstetrics/ providers characteristics and ANC provided to assess the differences in the mean of total ANC content scores. The full model analysis contained these independent variables: parity, risk level, clinic type, percentage of total visits attended by specific providers, and ANC utilisation adequacy.

To facilitate the grasp on adequacy of ANC content, we used categorical ANC content score—inadequate (≤ 79%) and adequate (≥ 80%)—in the assessment of ANC content level and pregnancy outcomes using binary logistic regression. This analysis at first performed a univariate analysis using pregnancy outcomes and ANC content adequacy; the analysis was subsequently controlled for ANC utilisation.

## Results and Discussion

### Respondent characteristics

The mean maternal age of the women at the first visit was 28.7 years. 56% had secondary education and 37% were tertiary educated. Only 4% were primary educated and less than 1% without any formal education. Mean parity was 1.2; nulliparous and multiparous were 37% and 63% respectively. Seventy-two percent were considered low-risk pregnancies, while 28% were high-risk. Thirty-four percent of the samples received their ANC at the clinics with 301–500 planned daily patient capacity, 47% at the clinics with 150–300 planned daily patient capacity, and 19% at the clinics with <150 planned daily patient capacity. Out of the total visits of the women, the mean percentage of service delivered by community nurse was 49%, by registered nurse with postgraduate qualification 28%, and by medical officers 46%. On average, nearly half of a pregnant woman’s total visits involved interaction with a community nurse and a medical doctor. In terms of ANC utilisation level, 21% had inadequate, 16% adequate and 63% “adequate-plus” utilisation.

The inventory of care providers showed that the clinics with planned daily workload of 301–500 and 150–300 patients assigned two medical officers to attend to maternal-child-health patients on a daily basis. These clinics also had an in-house Family Medicine Specialist whom the medical officers could refer the high-risk women. The clinics with planned daily workload below 150 had one medical officer responsible for maternal-child-health services, as well as access to Family Medicine Specialist on scheduled visiting basis. Total nursing staff for maternal-child-health services was approximately 35–40 for the clinics with 301–500 planned daily workload and around 25 staff for the clinics with 150–300 planned daily workload. The clinics with below 150 planned daily workload had around 8–9 nursing staff. In general, majority of the nursing staff were community nurses, ranging from around 40% to 60% of the total nursing staff. Staff nurses with postgraduate qualification constituted around 20–25% of the total nursing staff. Overall the findings revealed that the clinics from the same stratum had similar staffing. Personal communication with the nursing officers of these health clinics revealed no major issue concerning overall staff posting. It was informed that clinics with planned daily workload below 150, which were often located in less populated areas, generally faced more difficulty to attract staff nurses with post-graduate qualification.

### Extent of adherence to recommended antenatal care content

Fifty-two percent of the women had less than 80% of essential recommended ANC content documented in their records. This indicated that around half of the pregnant women did not receive adequate recommended care. The mean total score was around 77%. Assessment by ANC content components revealed that the mean score for PE, HS and CM components were similar at around 84–85% each; whereas HE component had the lowest score at only around 35%. The substantially lower score in health education echoed the finding of other studies in which health education was frequently less performed as compared to physical examination, screening, or prescription [4–6]. Other studies found 42% of the women were not informed of any pregnancy danger signs [23], and 30% to 55% of the women did not receive half of the recommended health education topics [9].

Table 1 showed that in the PE component, attention on oral hygiene or referral for oral health services is warranted, given that more than half of the pregnant women have no documented referral or advice provided. Likewise, physical examinations such as cardiovascular, respiratory and thyroid had lesser degree of documented compliance as compared to other examinations. Routine basic urine and blood screenings under HS component had almost universal adherence, except for Hepatitis-B screening. Although Malaysia guideline recommends Hepatitis-B screening at booking, none of the clinics surveyed performed the test or asked the pregnant women’s hepatitis status. Though it was recognised that antenatal hepatitis B screening is effective in reducing the risk of mother-to-child transmission [24–26], meeting the need of screening might be challenging for many health clinics since many are currently without the equipment and financial allocation to conduct this particular test. The cost benefit and screening strategy will need to be reviewed within the current health system context and epidemiology profile.

**Table 1:** Documented antenatal care content provided to pregnant women, mean of number of times performed, and percentage of pregnant women given care according to compliance criteria (n = 522).

Antenatal care content assessed	Mean	Care given, %	Care not given, %
**(I) PHYSICAL EXAMINATION (PE)**			
oral hygiene (or referral for oral health services)	0.61	44.4	55.6
general condition—pallor, cyanosis, varicose veins, etc.	-	99.6	0.4
cardiovascular system	1.65	60.7	39.3
respiratory	1.68	61.9	38.1
thyroid	1.42	45.2	54.8
abdomen—previous scar/ other masses	2.49	99.4	0.6
height	NA	97.9	2.1
weight	9.55	98.3	1.7
blood pressure	9.56	98.3	1.7
breast	1.97	92.5	7.5
symphysis-fundal height	8.29	96.4	3.6
foetal lie and presentation	8.32	97.7	2.3
foetal heart auscultation	8.06	97.9	2.1
oedema	9.18	94.1	5.9
**(II) HEALTH SCREENING (HS)**			
urine protein	9.08	96.7	3.3
urine sugar	9.06	96.7	3.3
Haemoglobin or FBC	6.70	99.6	0.4
ABO blood grouping	0.98	98.1	1.9
Rhesus factor blood test	0.98	98.1	1.9
VDRL	0.98	98.1	1.9
HIV	0.96	96.4	3.6
Ultrasound, abdominal (≥ two times)	2.61	78.4	21.6
Hepatitis B	0	0	0
**(III) CASE MANAGEMENT (CM)**			
routine medical examination by doctor- 1^st^	NA	97.7	2.3
routine medical examination by doctor- 2^nd^	NA	92.0	8.0
risk assessment according to schedule	2.85	35.4	64.6
appropriate risk tagging	NA	95.2	4.8
ultrasound performed before or at 24 weeks of pregnancy	NA	82.2	17.8
immunisation—anti-tetanus vaccination (in dose)	1.33	96.2	3.8
haematinic supplement (include folic acid or multivitamins supplements)	6.72	90.2	9.8
**(IV) HEALTH EDUCATION (HE)**			
nutritional/dietary advice—antenatal	5.42	99.2	0.8
nutritional/dietary advice—postnatal/ breastfeeding	0.01	1.3	98.7
recommendations for family planning/ contraception	1.55	66.7	33.3
preparation for birth	1.44	73.9	26.1
birth process (sign & symptom and related advice)	2.18	84.3	15.7
common discomfort during pregnancy and solutions	0.25	22.6	77.4
recommendations for breastfeeding	1.17	71.3	28.7
common disorders in pregnancy (at least 2 topics below):	-	26.1	73.9
pregnancy induced hypertension	0.03	-	-
preeclampsia/ impending eclampsia	0.31	-	-
gestational diabetes mellitus	0.18	-	-
anaemia	0.52	-	-
bleeding during pregnancy	0.47	-	-
early booking	0.06	6.1	93.9
foetal development	0.17	12.8	87.2
exercise antenatal/ postnatal	0.03	3.3	96.7
newborn care, baby bathing	0.00	0.4	99.6
jaundice baby care	0.20	19.7	80.3
postnatal care	0.06	5.2	94.8

The mean of risk assessment performed for each woman was 2.9 times; only 35% were assessed according to the complete risk assessment criteria which were adjusted for gestational period of initiation and birth (S1 Table). The current risk assessment and coding system consists of an extensive list of conditions. While it is acknowledged that this risk assessment system has been useful as a management tool as it enables the nursing staff to refer or admit a pregnant woman with problem swiftly, it had also been commented on the need to streamline the assessment system in order to optimise the time spent on coding [20]. It is of the opinion that the current assessment system may be reviewed to streamline and focus on risk factors that have been found to be associated with adverse maternal and child outcomes; for example, maternal morbidity and mortality, preterm birth and stillbirth outcomes. It will be useful to assess the current risk factors included in the assessment system in terms of their benefit and influence in the delivery of ANC. It is also worthwhile to continue assessing the profile of the women who passed the risk assessment and classified as no- or low-risk, but had adverse pregnancy outcome. This may shed some lights concerning the aetiologies of these outcomes, particularly preterm birth and stillbirth, that are known to differ by gestational age, genetics, and environmental factors [27].

The MOH Malaysia recommends performing abdominal ultrasound before 24 weeks [19]. This study found that the mean gestation age for the first ultrasound was around 18 weeks. 18% of the women did not have their first ultrasound at or before 24 weeks (Table 1). The mean ultrasound (including those done by other providers) was 2.6. Around twenty-two percent of pregnant women had less than 2 ultrasounds (Table 1).

Further analysis of this present study showed that the checklist for health advice, which was the basis for the assessment on health advice provided, was rarely completed. Majority (45%) of the women’s records did not fill this checklist, while 37% of the records had it partially filled. Provision of predefined health advice topics was not adhered to. Pregnant women were often advised on similar topics during their visits. For example, the mean for the number of times antenatal dietary advice given was 5.4. Another common advice given was adequate rest and sleep (mean 2.7), which was not part of the health advice topics in the checklist. In contrast, advice on physical exercise which is part of the checklist was rarely given (2.9%). Advice on postnatal care was also seldom given (Table 1). Repeated advice could be due to low compliance or persistent conditions that warranted similar advice. Overall, it appeared that different topics are attached with different importance by the nurses, but reasons for that were not investigated in this study and should be explored further.

### Period of gestation when selected time-appropriate examinations were initiated

On average, examinations for symphysis-fundal height, foetal presentation, and foetal heart auscultation were commenced at around 18–19 gestation weeks (Table 2). Half of the women were examined on these parameters by 18 weeks and earlier. In comparison, the guidelines [19] recommend examining symphysis-fundal height at 22 weeks onwards. Though earlier examination as found in this study does not have harmful effect, earlier initiation has no proven benefit to ANC, but has economic implication on provider side since the staff will spend more time on the women unnecessarily. Similarly, the local guidelines [19] recommend examining foetal presentation from 32 weeks. A much early start as found in this study will have substantial economic/resource implication on provider side without the due benefit, and may cause unnecessary worry to the women.

**Table 2:** Period of gestation when selected examinations were initiated (n = 522).

Period of gestation when examination initiated, gestational weeks	Mean	Median	SD	Recommended initiation according to guidelines, gestational weeks [19]
symphysis-fundal height	18.3	18.0	4.2	22
foetal lie/ presentation	18.6	18.0	4.0	32
foetal heart auscultation	19.6	19.0	3.7	24 (pinard) or 14 (Doppler)

### Factors associated with ANC content score

GLM univariate analysis showed that risk level of pregnancy (P = 0.002), clinic type (P<0.001), and ANC utilisation level (P<0.001) were significantly associated with the mean ANC content score statistically. Parity and percentage of total visits attended by specific providers showed non-significant difference in the mean ANC content scores (P>0.05, Table 3). The mean content score among the low-risk women was 78%, while the mean score among the high-risk was 75% (p = 0.002). The actual difference, albeit significant, is small, and may be limited to a few care items. There has not been much comparison on the extent of adherence to ANC content by the risk level of pregnant women. Study conducted in developed setting showed a higher proportion of high-risk women received ≥ 80% of documented ANC content than low-risk women (34% vs 24%) [7]. This is different from the finding of this study that recorded a lower proportion of high-risk women received ≥ 80% of recommended ANC content than the low-risk (40% vs 52%). During the review of the records, we found that the ANC check-up and medical consultation of the high-risk cases appeared to focus on the high-risk condition, and less on other aspects of care. For example, the care and advice given to a pregnant woman with gestational diabetes mellitus would heavily focus on blood sugar monitoring, but lesser attention on other aspects. While it is understandable to focus on a particular risk condition, it is however not desirable to forgo other general aspects of care a pregnant woman would also need. This reflects the notion of treating a patient (pregnant woman with medical condition) as “a sum of the parts”, as opposed to the call for a more holistic approach that should be concerned with the multidimensional needs of the women and not only with their biological care [28–30].

**Table 3:** Difference of mean for antenatal care content score in % by selected characteristics (n = 522).

Characteristics	Mean antenatal care content score	p
Parity:		0.584
Nullipara	76.5	
Multipara	76.2	
Risk level of pregnancy:		0.002
Low-risk	77.6	
High-risk	75.1	
Clinic type by planned daily patients capacity:		<0.001
below 150	78.4	
150–300	74.3	
301–500	76.3	
*Pairwise comparison*:		
*Below 150 and 150–300*		*<0*.*001*
*Below 150 and 301–500*		*0*.*051*
*150–300 and 301–500*		*0*.*006*
ANC utilisation level:		<0.001
Inadequate	76.3	
Adequate	74.6	
Adequate-plus	78.1	
*Pairwise comparison*:		
*Inadequate and adequate-plus*		*0*.*048*
*Inadequate and adequate*		*0*.*239*
*Adequate and adequate-plus*		*<0*.*000*
% total visits attended by community nurse	-	0.459
% total visits attended by staff nurse with postgraduate qualification	-	0.313
% total visits attended by medical officer	-	0.322

GLM Univariate full model analysis containing parity, risk level, clinic type, percentage of total visits attended by specific providers, and antenatal care utilisation adequacy.

The mean ANC content score among the clinics with daily capacity of below 150 patients was 78%, significantly higher than the mean ANC content score among the bigger clinics with daily capacity of 150–300 patients statistically (74%, P<0.001), while the difference with the clinics with 301–500 daily capacity was not statistically significant (76%, P = 0.051). The clinics with 150–300 daily capacity had a lower mean ANC content score compared to the clinics with 301–500 daily capacity, though the difference is small (P = 0.006). The finding is consistent with other studies that found adherence to ANC content differed among provider sites [4, 6, 7, 31]. In the context of this study, the staffing norm of a clinic is proportional to the planned daily capacity of the clinic. Finding from this study showed the number of nursing staff from the biggest to the smallest clinics were approximately 35–40, 25 and 8–9 respectively. Both the smallest clinics in the below 150 patients strata were located in the less populated districts. Based on the number of clinics and total population in the districts [32], the average population coverage for these two smallest clinics ranged from around 20,000 to 30,000 per clinic. In contrast, the other four bigger clinics were located in the most populated districts. Average population coverage per clinic was proportionally much higher, ranged from about 130,000 to 300,000 people, much higher than the planned target ratio of 1:20,000 [12]. In comparison, the population coverage for the four bigger clinics was approximately 5 to 10 times higher than the two smallest clinics. Despite the much higher population coverage among the four bigger clinics, the nurse ratio between the clinics in the 301–500 patients strata and the smallest clinics in the <150 patients strata was around 4 times higher; and the nurse ratio between the clinics in the 150–300 patients strata and the smallest clinics <150 patients strata was only around 3 times higher. This implies the presence of a higher actual user load than planned capacity, resulted in higher provider-user ratio, and thus reduced provider-user interaction time. It had been acknowledged that there is a shortage of health clinics in the densely populated areas such as the Klang Valley which includes the densely populated study districts where the four bigger clinics were located. The overall population ratio for MOH health clinics of 1:33,600 has not met the target of 1:20,000 [12].

The mean content score among women in the “adequate” ANC utilisation category as defined by the modified Adequacy of Prenatal Care Utilisation Index was 75%, significantly lower than the mean content score among women in the “adequate-plus” ANC utilisation category statistically (78%, P<0.001). The difference of the content score between the women in “inadequate” and “adequate” utilisation category was not significant statistically (P = 0.239). The difference between women with “inadequate” and “adequate-plus” utilisation was very small, although statistically significant (P = 0.048). The slightly lower mean content score among the women in “adequate” utilisation category compared to “adequate-plus” utilisation category was largely related to the scoring criteria for the ANC content assessment (S1 Table). Majority of the items in PE, HS and CM components were adjusted for the period of gestation at initiation and birth, except for HE component. As a result, having utilisation level above recommended which also implies higher number of ANC visits than recommended, presents more opportunities for these women to be given more health advice than women with adequate level of utilisation. Nevertheless, utilisation level higher than recommended should not be encouraged especially among the low-risk pregnancies. Studies have concluded that antenatal care for women without risk could be provided with fewer visits [33, 34]. Delivery of ANC should aim for completeness of care within the recommended schedule.

### Antenatal care content and pregnancy outcomes

Table 4 presents the pregnancy outcomes by adequacy level of ANC content. Based on the observed significance level, ANC content adequacy was significantly associated with preterm birth statistically, unadjusted and adjusted for ANC utilisation. The odds of preterm birth in the inadequate category were nearly four times that of the adequate category, adjusted for ANC utilisation. Interpretation of this finding has to consider the scoring criteria for the HE component and a few other items which were not adjusted for gestation age at birth as explained earlier, as well as the ANC utilisation index that was adjusted for gestation age at birth. A woman who had preterm birth and categorised as having adequate ANC utilisation level would have fewer visits than a women who had term birth and categorised as having adequate ANC utilisation level. As such, the preterm birth woman would have lesser opportunities to receive some of the care items, for example health advice, and thus had lower ANC content score.

**Table 4:** Antenatal care content adequacy and pregnancy outcomes (n = 522).

Antenatal care content category	Preterm birth (n = 36)	Low birth weight (n = 66)	Stillbirth (n = 16)	Combined outcomes (n = 86)
	**Crude OR (95% CI)**			
Inadequate	3.53 (1.58–7.90)	1.22 (0.72–2.04)	0.72 (0.26–1.96)	1.44 (0.90–2.30)
Adequate	1.00	1.00	1.00	1.00
	* **Adjusted OR (95% CI)**			
Inadequate	3.83 (1.70–8.63)	1.29 (0.76–2.18)	0.76 (0.28–2.07)	1.54 (0.96–2.48)
Adequate	1.00	1.00	1.00	1.00

* odds ratios adjusted for antenatal care utilisation

Nevertheless, this may also be due to lesser opportunities to receive some of the care items that may aid in prevention of preterm birth. Adequate level of ANC content provided to the women may contribute to early detection and timely management of risk for preterm birth. Studies have found that lack of educating pregnant women on the signs and symptoms of preterm labour as well as advice to call the health provider was associated with around three times higher risk of preterm birth [35]. The knowledge on recognising preterm risk, possible causes, and action to take will better prepare the pregnant women to seek medical care promptly should the situation arise. Furthermore, routine ANC examination or screening might help to detect and promptly manage risk conditions such as bacteriuria or placenta praevia. ANC content did not appear to influence the LBW and stillbirth outcomes. The combined outcomes showed the odds of adverse birth outcomes in the inadequate category were around 1.5 times that of the adequate category, controlled for ANC utilisation.

### Limitations of study

Our study encountered limitations associated with retrospective study using medical records, i.e. evidence of care rendered and quality of data. The evidence of care rendered and the quality of data collected are based on the quality of medical documentation. It is assumed that all elements of care rendered were fully documented, which might not be necessarily true. For the purposes of data analysis, no entries will be interpreted as “care/intervention not rendered”. This may result in a slight under-estimation of the proportion of pregnant women on whom each of the intervention was performed since the resulting estimate did not include those on whom the interventions were actually performed but not documented. Nevertheless, essential events or procedures were generally documented. In addition, each record was carefully examined for evidence of care rendered, but was not documented in the care notes. Future studies may include an independent validation study to estimate the recording bias. For example, observe a subsample of patient-provider interactions and compare with the records; this will give an estimate of the recording bias, although there might be reactive effects due to the presence of the observer.

The ANC content assessment conducted by this study was based on the current national ANC guidelines. In comparison with the evidence-based guidelines from other developed countries (the United Kingdom, Australia, and the United States) with better maternal and child health indicators, in particular United Kingdom and Australia, the Malaysia guidelines might have some rooms for improvement that could be carefully examined and addressed. Future study might consider assessing the ANC content using an “improved” guideline that would address the gaps identified.

## Conclusions

The study shows that half of the women had less than 80% of the recommended ANC content documented. Examining the extent of adherence to recommended practices underscores areas for improvement, notably when these practices are assessed in terms of initiation timing and frequency of interventions. Delivery of ANC should consider appropriate timing of initiation due to resource implication and maternal wellbeing. Provision of health advice which scored poorly in this study at only 35% mean score is a concern considering the importance of health education in influencing the behaviour and outcome of pregnant women. Delivery of the health topics needs to be monitored and adhered to ensure each woman has adequate coverage for essential health education. Moreover, it appeared that different health advice topics were attached with different importance by the nurses because some topics were more frequently provided to the women than others. Future studies could explore the reasons for this observation. High-risk pregnancies do require additional care and medical attention for the high-risk conditions. The high-risk women however still require basic ANC to identify or prevent other complications; delivery of ANC needs to move away from focusing on specific biological care. It is important to advocate that all pregnant women be given the complete scope of basic ANC regardless of their risk level. Our study shows that the extent of adherence to ANC content was associated with clinic-population ratio of a clinic. The staffing norm of a clinic is proportional to the planned capacity of the clinic; it is however essential to provide additional resource in highly populated areas to ensure adequate provider-user ratio.

ANC content adequacy was significantly associated with preterm birth; having inadequate ANC content was associated with higher prevalence of preterm birth. This may be due to lower number of ANC visits associated with preterm births and thus lesser opportunities to receive some of the care that resulted in lower content score. However, this may also be due to lesser opportunities to receive care that may mitigate the risk of preterm birth. Having adequate level of ANC content may contribute to early detection and timely management of risk for preterm birth. In essence, the current practice of ANC and the assessment conducted by this study on ANC content provided to pregnant women were based on the current national ANC guidelines. In comparison with the recommended guidelines from other developed countries with better maternal and childbirth indicators, the local guidelines might have rooms for improvement that should be carefully examined.

## Supporting Information

S1 Table Minimal Requirements for Recommended Antenatal Care Content and Compliance Criteria for Scoring. (DOCX)

S2 Table Supporting Data. (DOCX)
