# Supplementary material for: Assessing the Extent of Adherence to the Recommended Antenatal Care Content in Malaysia: Room for Improvement
Source: PLoS One. 2015 Aug 13;10(8):e0135301. doi: 10.1371/journal.pone.0135301 (PMC4536216; doi:10.1371/journal.pone.0135301)
Supplement: S2 Table — (DOCX) [file pone.0135301.s002.docx]

Table S2.

Supporting Data: Respondents Characteristics

| **Characteristics** |  | **Unit** | **Clinic category by expected daily workload** | | | **Total** |
| --- | --- | --- | --- | --- | --- | --- |
|  |  |  | **301-500** | **150-300** | **below 150** |  |
| **maternal age at first visit** | <=19 | n (%) | 3 (0.6%) | 4 (0.8%) | 4 (0.8%) | 11 (2.1%) |
|  | 20-34 | n (%) | 151 (28.9%) | 210 (40.2%) | 78 (14.9%) | 439 (84.1%) |
|  | >=35 | n (%) | 23 (4.4%) | 33 (6.3%) | 16 (3.1%) | 72 (13.8%) |
|  | Total | n (%) | 177 (33.9% | 247 (47.3%) | 98 (18.8%) | 522 (100.0%) |
|  | Mean (SD) |  |  |  |  | 28.7 (5.0) |
| **ethnicity** | Malay | n (%) | 121 (23.2%) | 198 (37.9%) | 77 (14.8%) | 396 (75.9%) |
|  | Chinese | n (%) | 26 (5.0%) | 22 (4.2%) | 19 (3.6%) | 67 (12.8%) |
|  | Indian | n (%) | 25 (4.8%) | 18 (3.4%) | 1 (0.2%) | 44 (8.4%) |
|  | Indigenous people | n (%) | 5 (1.0%) | 9 (1.7%) | 1 (0.2%) | 15 (2.9%) |
|  | Total | n (%) | 177 (33.9%) | 247 (47.3%) | 98 (18.8%) | 522 (100.0%) |
| **maternal education** | No formal education | n (%) | 3 (0.6%) | 1 (0.2%) | 0 (0.0%) | 4 (0.8%) |
|  | Primary | n (%) | 6 (1.1%) | 7 (1.3%) | 6 (1.1%) | 19 (3.6%) |
|  | Secondary | n (%) | 91 (17.4%) | 139 (26.6%) | 64 (12.3%) | 294 (56.3%) |
|  | Tertiary (certificate or diploma) | n (%) | 39 (7.5%) | 54 (10.3%) | 18 (3.4%) | 111 (21.3%) |
|  | Tertiary (advanced diploma, degree or higher) | n (%) | 31 (5.9%) | 42 (8.0%) | 9 (1.7%) | 82 (15.7%) |
|  | unknown | n (%) | 7 (1.3%) | 4 (0.8%) | 1 (0.2%) | 12 (2.3%) |
|  | Total | n (%) | 177 (33.9%) | 247 (47.3%) | 98 (18.8%) | 522 (100.0%) |
| **maternal occupation** | Legislators, senior officials and managers | n (%) | 4 (0.8%) | 3 (0.6%) | 0 (0.0%) | 7 (1.3%) |
|  | Professional | n (%) | 19 (3.6%) | 28 (5.4%) | 10 (1.9%) | 57 (10.9%) |
|  | Technicians and associate professionals | n (%) | 23 (4.4%) | 33 (6.3%) | 11 (2.1%) | 67 (12.8%) |
|  | Clerical workers | n (%) | 35 (6.7%) | 41 (7.9%) | 11 (2.1%) | 87 (16.7%) |
|  | Service workers, shop and market sales workers | n (%) | 22 (4.2%) | 23 (4.4%) | 18 (3.4%) | 63 (12.1%) |
|  | Craft and related trades workers | n (%) | 2 (0.4%) | 0 (0.0%) | 0 (0.0%) | 2 (0.4%) |
|  | Plant and machine operators and assemblers | n (%) | 5 (1.0%) | 21 (4.0%) | 2 (0.4%) | 28 (5.4%) |
|  | Elementary occupations | n (%) | 5 (1.0%) | 2 (0.4%) | 1 (0.2%) | 8 (1.5%) |
|  | Others - housewives, students, unemployed | n (%) | 60 (11.5%) | 94 (18.0%) | 45 (8.6%) | 199 (38.1%) |
|  | unknown | n (%) | 2 (0.4%) | 2 (0.4%) | 0 (0.0%) | 4 (0.8%) |
|  | Total | n (%) | 177 (33.9%) | 247 (47.3%) | 98 (18.8%) | 522 (100.0%) |
| **spouse’s occupation** | Legislators, senior officials and managers | n (%) | 5 (1.0%) | 7 (1.3%) | 3 (0.6%) | 15 (2.9%) |
|  | Professional | n (%) | 17 (3.3%) | 26 (5.0%) | 7 (1.3%) | 50 (9.6%) |
|  | Technicians and associate professionals | n (%) | 53 (10.2%) | 73 (14.0%) | 24 (4.6%) | 150 (28.7%) |
|  | Clerical workers | n (%) | 14 (2.7%) | 17 (3.3%) | 4 (0.8%) | 35 (6.7%) |
|  | Service workers, shop and market sales workers | n (%) | 28 (5.4%) | 50 (9.6%) | 19 (3.6%) | 97 (18.6%) |
|  | Skilled agricultural and fishery workers | n (%) | 0 (0.0%) | 0 (0.0%) | 16 (3.1%) | 16 (3.1%) |
|  | Craft and related trades workers | n (%) | 7 (1.3%) | 5 (1.0%) | 9 (1.7%) | 21 (4.0%) |
|  | Plant and machine operators and assemblers | n (%) | 31 (5.9%) | 53 (10.2%) | 12 (2.3%) | 96 (18.4%) |
|  | Elementary occupations | n (%) | 9 (1.7%) | 4 (0.8%) | 2 (0.4%) | 15 (2.9%) |
|  | Armed forces | n (%) | 3 (0.6%) | 7 (1.3%) | 1 (0.2%) | 11 (2.1%) |
|  | Others | n (%) | 0 (0.0%) | 1 (0.2%) | 1 (0.2%) | 2 (0.4%) |
|  | unknown | n (%) | 10 (1.9%) | 4 (0.8%) | 0 (0.0%) | 14 (2.7%) |
|  | Total | n (%) | 177 (33.9%) | 247 (47.3%) | 98 (18.8%) | 522 (100.0%) |
| **gravidity** | Primigravida (1) | n (%) | 69 (13.2%) | 72 (13.8%) | 33 (6.3%) | 174 (33.3%) |
|  | Multigravida (=>2) | n (%) | 108 (20.7%) | 175 (33.5%) | 65 (12.5%) | 348 (66.7%) |
|  | Total | n (%) | 177 (33.9%) | 247 (47.3%) | 98 (18.8%) | 522 (100.0%) |
|  | Mean (SD) |  |  |  |  | 2.4 (1.5) |
| **parity** | Nulliparous (0) | n (%) | 77 (14.8%) | 82 (15.7%) | 36 (6.9%) | 195 (37.4%) |
|  | Multiparous (=>1) | n (%) | 100 (19.1%) | 165 (31.6%) | 62 (11.9%) | 327 (62.6%) |
|  | Total | n (%) | 177 (33.9%) | 247 (47.3%) | 98 (18.8%) | 522 (100.0%) |
|  | Mean (SD) |  |  |  |  | 1.2 (1.3) |
| **tag colour (white or coloured)** | white tag | n (%) | 62 (11.9%) | 75 (14.4%) | 23 (4.4%) | 160 (30.7%) |
|  | coloured tag | n (%) | 115 (22.0%) | 172 (33.0%) | 75 (14.4%) | 362 (69.3%) |
|  | Total | n (%) | 177 (33.9%) | 247 (47.3%) | 98 (18.8%) | 522 (100.0%) |
| **risk code** | white | n (%) | 62 (11.9%) | 75 (14.4%) | 23 (4.4%) | 160 (30.7%) |
|  | green | n (%) | 62 (11.9%) | 107 (20.5%) | 46 (8.8%) | 215 (41.2%) |
|  | yellow | n (%) | 51 (9.8%) | 62 (11.9%) | 27 (5.2%) | 140 (26.8%) |
|  | red | n (%) | 2 (0.4%) | 3 (0.6%) | 2 (0.4%) | 7 (1.3%) |
|  | Total | n (%) | 177 (33.9%) | 247 (47.3%) | 98 (18.8%) | 522 (100.0%) |
| **adequacy of ANC utilisation** | Inadequate | n (%) | 45 (8.6%) | 46 (8.8%) | 16 (3.1%) | 97 (18.6%) |
|  | Adequate | n (%) | 36 (6.9%) | 38 (7.3%) | 11 (2.1%) | 95 (18.2%) |
|  | Adequate-plus | n (%) | 96 (18.4%) | 163 (31.2%) | 71 (13.6%) | 330 (63.2%) |
|  | Total | n (%) | 177 (33.9%) | 247 (47.3%) | 98 (18.8%) | 522 (100.0%) |
| **adequacy of ANC content** | Inadequate (<80%) | n (%) | 92 (17.6%) | 145 (27.8%) | 33 (6.3%) | 270 (51.7%) |
|  | Adequate (>80%) | n (%) | 85 (16.3%) | 102 (19.5%) | 65 (12.5%) | 252 (48.3%) |
|  | Total | n (%) | 177 (33.9%) | 247 (47.3%) | 98 (18.8%) | 522 (100.0%) |
